# 

**Published:** 1888-09

**Authors:** 


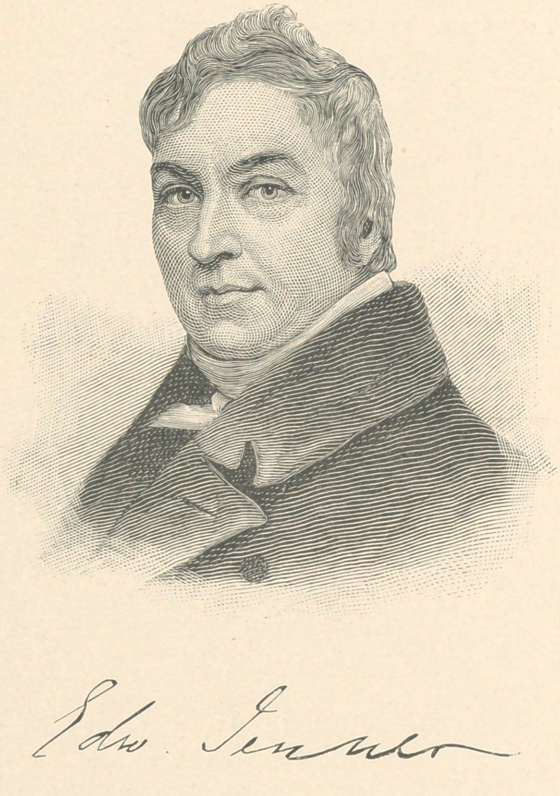

EDWARD JENNER, M. D., LL.D., F. R. S.

"My opinion of vaccination is precisely as it was when I first promulgated the discovery. It is not in the least strengthened by any event that has happened, for it could gain no strength; it is not in the least weakened, for if the failures you speak of had not happened, the truth of my assertions respecting those coincidences which occasioned them would not have been made out."

### BOOKS RECEIVED.

Henry's Posological Tables.

Transactions of the New York State Medical Association. 1887.

Medical Publications Harvard Medical School. 1887.

Fourteenth Annual Report of the Secretary of the State Board of Health of Michigan.

A System of Obstetrics by American Authors. Vol. I.

Annual of the Universal Medical Sciences. Issue of 1888. Charles E. Sajons, M. D., editor.

Practical Electro-Therapeutics. By William F. Hutchinson, M. D.

Manual for Hospital Nurses. By E. J. Domville, M. D.

Theine in the Treatment of Neuralgia. By Thomas J. Mays, M. D.

A Guide to the Practical Examination of the Urine. Sixth edition. By James Tyson, M. D.

The Applied Anatomy of the Nervous System. By Ambrose L. Ranney, A. M., M. D.

Reference Hand-Book of the Medical Sciences. Vol. VI. Edited by A. H. Buck, M. D.

A Practical Text-Book on the Diseases of Women.

By Arthur H. N. Lewers, M. D., Lond., M. R. C. P. Lond.

Anæsthetics; Their Use and Administration. By D. W. Burton, M. D., B. S.

A Clinical Atlas of Venereal and Skin Diseases. Parts I and II. By Robert W. Taylor, A. M., M. D.

Comparative Studies of Mammalian Blood. By Henry F. Formad, B. M., M. D.

The National Formulary of Unofficinal Preparations.

Atlas of Venereal and Skin Diseases. Fasciculi V and VI. By P. A. Morrow, A. M., M. D.

Intra-Cranial Tumors. By Byrom Bramwell, M. D., F. R. C. P. E., F. R. S. E.

### PAMPHLETS RECEIVED.

Water: Its Impurities Gathered from the Air and Earth. By C. W. Moore, M. D.

The Orthopedic Treatment of Paralysis of the Anterior Muscles of the Thigh. By A. B. Judson, M. D.

On Exercise for Prevention and Cure of Deformities. By A. H. P. Leuf, M. D.

Vesico-Vaginal Fistula. By Reuben A. Vance, M. D.

Effects of Food Preservatives on the Action of Diastase, Pancreatic Extract, and Pepsin. By Henry Leffman, M. D., and Wm. Beam, M. A.

The Intra-Uterine Stem in the Treatment of Flexions. By A. Reeves Jackson, A. M., M. D.

The Causation of Pneumonia. By Henry B. Baker, M. D.

Die Entzündung der Peripheren Nerven, deren Pathologie und Behandlung. By E. Leyden.

Some Remarks on Opening the Mastoid Process. By Frank Allport, M. D.

An Operation for Simple Forms of Eutropium. By Frank Allport, M. D.

Rectal Insufflation of Hydrogen Gas. By N. Senn, M. D., Ph. D.

An Experimental Contribution to Intestinal Surgery. By N. Senn, M. D., Ph. D.

Fracture of the Sternum, with Dislocation of the Fragments. By B. J. D. Irwin, M. D.

Some Retrospective and Prospective Thoughts on Surgery. By Donald McLean, M. D.

Cocaine Dosage and Cocaine Addiction. By J. B. Mattison, M. D.

Tornado Circular No. 1. Signal Service, War Department.

Camp Hygiene. Pennsylvania State Board of Health.

Transactions of the Massachusetts Medico-Legal Society, Vol. I. No. 10.

Proceedings of the State Sanitary Convention of Pennsylvania. May 12 to 14, 1886.

Report of Proceedings of the Illinois State Board of Health. Quarterly Meeting April 19, 20, 1888.

Annual Report of the Department for the Insane of the Pennsylvania Hospital.

Report of the Department of Health of the City of Chicago, for the year 1887.

### ANNOUNCEMENTS.

### CONGRESS OF AMERICAN PHYSICIANS AND SURGEONS.

Circular No. III.

Wshington, D. C., July, 1888.

The Committee of Arrangements takes pleasure in announcing to the members and invited guests of the special societies taking part in the Congress that the arrangements are sufficiently advanced to assure the success of the First Triennial Session of the Congress of American Physicians and Surgeons, which will be held in the city of Washington during the 18th, 19th, and 20th of September next.

A number of distinguished physicians and surgeons have signified their acceptance of the invitation to attend, among whom may be named Sir Spencer Wells, Sir Andrew Clark, Sir William McCormac, Drs. W. O. Priestley, William Ord, and Grainger Stewart; Mr. Lawson Tait, Mr. Victor Horsley, Mr. Thomas Bryant, Mr. Thomas Annandale; Professors Ferrier, Esmarch, and Gerhardt; Drs. Rafael Lavista, of Mexico; J. L. Reverdin, of Geneva; O. W. Holmes and H. I. Bowditch, of Boston; Joseph Leidy, of Philadelphia; W. Kingston and Eccles, of Canada.

The preliminary programmes of the participating societies have been published. It is hoped that the classified and final programmes will be forwarded without further delay. Several are already in possession of the committee. As soon as all are received, the final programme of the Congress will be printed and distributed.

Places of meeting for the Congress and each of the societies have been secured, conveniently located, so that members may interchange attendance without annoyance.

The meetings of the Congress will be held during the evenings, beginning at 8 o'clock p. m.; on the evenings of the 18th and 19th the meetings will be held in the main hall of the Grand Army Building, 1412 and 1414 Pennsylvania Avenue, and on the last (Thursday evening) in the hall of the National Museum. During this evening the Army Medical Museum and Library Building, alongside of the Museum Building, will be lighted and opened for the inspection of the members and invited guests. The meetings of the societies will be held during the day, according to the programme each may respectively provide. The sessions will be open to the profession.

On Monday evening, September 17, a dinner will be given by members of the Congress to the guests of the participating societies. Invitations to this dinner will be sent only to the specially invited guests who have indicated their acceptance. The contributing members will receive cards of admission. It will be limited exclusively to members of the Congress and invited guests.

An informal collation will be served at Willard's Hotel on Tuesday evening, after the adjournment of the meeting of the Congress, to the guests and those members who may choose to attend. A similar entertainment will be served in the National Museum Building on Thursday night, after the final adjournment of the Congress.

Guests are requested to notify the chairman immediately after their arrival in Washington, giving their address, and stating whether they have ladies with them. Special arrangements will be made for the entertainment of the wives and daughters of the guests.

Hotel accommodations are ample, and conveniently located to the places of meeting.

The secretaries of the special societies are requested to forward to the chairman the names and addresses of their foreign guests.

Members of the Congress and their guests are expected to register. A parlor in Willard's Hotel will be provided for that purpose, from which the mail of the members and guests will be distributed, and at which the city residence of each member or guest can be ascertained.

All communications should be addressed to the chairman of the committee,

SAMUEL C. BUSEY, M. D.,
1545 *I St. N. W., Washington City,*
*Chairman Committee of Arrangements.*

---

THE American Orthopedic Association will meet in Washington, D. C., on September 18, 19, and 20.

THE next meeting of the American Rhinological Association will be held in Cincinnati, Ohio, in September.

---

THE fifth annual meeting of the American Climatological Association will be held in Washington, D. C., September 18, 19, and 20, 1888.

---

THE annual meeting of the Southern Surgical and Gynæcological Association will be held at Birmingham, Alabama, on September 11, 12, and 13, 1888.

---

THE twelfth annual meeting of the American Dermatological Association will be held at Willard's Hotel, Washington, D. C., September 18, 19, and 20.

---

AT the annual meeting of the American Otological Society, held at the Pequot House, New London, Connecticut, on Tuesday, July 17, 1888, the Society voted to adjourn till 11 a. m. of Tuesday, September 18, 1888, at the Arlington Hotel, Washington, D. C.

---

AT the annual meeting of the AMERICAN OPHTHALMOLOGICAL SOCIETY, held at the Pequot House, New London, Connecticut, on Wednesday, July 18, 1888, the Society voted to adjourn till 11 a. m. of Wednesday, September 19, 1888, at the Arlington Hotel, Washington, D. C., to take part in the Congress of American Physicians and Surgeons.

Volume LVII.] SEPTEMBER, 1888. [Number 3.

### The CHICAGO MEDICAL JOURNAL AND EXAMINER

EDITOR:

S. J. JONES, M.D., LL.D

COLLABORATORS:

CHICAGO:

Professor E. ANDREWS, M.D., LL.D.
Professor J. BARTLETT. M.D.
Professor W. T. BELFIELD. M.D.
Professor F. BILLINGS, M.D.
Professor W. H. BYFORD. A.M., M.D.
Professor L. CURTIS. A.M., M.D.
Professor N. S. DAVIS, Jr., A.M., M.D.
Professor O. C. DeWOLF, A.M., M.D.
Professor E. C. DUDLEY, A.B., M.D.
Professor C. FENGER, M.D.
Professor J. N. HYDE, A.M., M.D.
Professor E. F. INGALS. A.M., M.D.
Professor F. S. JOHNSON, A.M., M.D.
Professor H. A. JOHNSON M.D., LL.D.
Professor C. W. PURDY, M.D.
Professor C. G. SMITH, A.M., M.D.
Professor E. WING, A.M., M.D

MILWAUKEE, WIS.:

Professor N. SENN, M.D., Ph. D.

WASHINGTON, D. C.:

S. O. RICHEY, M.D

UNITED STATES ARMY.
SURGEON D. L. HUNTINGTON, M.D.

UNITED STATES NAVY:

SURGEON-GENERAL J. M. BROWNE, M.D.

MEDICAL-DIRECTOR A. L. GIHON, A.M., M.D.

MARINE HOSPITAL SERVICE:
SURGEON S. T. ARMSTRONG, M.D., Ph.D.

Rand, McNally & Company, Printers.

1888.

